# TRPV1-target drugs for the treatment of orofacial pain

**DOI:** 10.3389/fphar.2025.1568109

**Published:** 2025-04-24

**Authors:** Ana Cláudia Macedo Andrade, Natalia Molina Esquivel, Florencia Goldschmied Rossel, Bruna Benso

**Affiliations:** School of Dentistry, Faculty of Medicine, Pontificia Universidad Catolica de Chile, Santiago, Chile

**Keywords:** pain, orofacial pain, toothache, facial neuralgia, TRPV cation Channels, vanilloid Receptor

## Abstract

Orofacial pain, encompassing sensory and emotional discomfort in the facial and oral regions, is a multifaceted condition that significantly impacts patients’ quality of life. This review focuses on the role of Transient Receptor Potential Vanilloid 1 (TRPV1) channels in modulating orofacial pain and new ligands targeting this receptor. TRPV1 channels act as key mediators of nociception, responding to stimuli such as temperature, pH changes, and capsaicin molecules. Recent advancements in TRPV1-targeted therapeutics, including natural, synthetic, and protein-based molecules, offer promising strategies for pain management. This review analyzed studies related to TRPV1-mediated pain inhibition, including seven clinical trials and preclinical investigations. The compounds studied in these works demonstrated pain relief, although adverse effects were reported. TRPV1-targeted molecules represent a novel avenue for developing innovative pharmacological interventions, addressing the limitations of current therapies, and improving patient outcomes in managing orofacial pain.

## 1 Introduction

Orofacial pain corresponds to unpleasant sensory and emotional experiences associated with the soft tissue of the head, face, and oral cavity (Labanca et al., 2023a). The term includes different local conditions, from dental to temporomandibular, neuropathic, burning mouth, chronic idiopathic facial pain, and headaches (da Silva Araújo Júnior et al., 2021). The assessment, diagnosis, and management of orofacial pain may be associated with high healthcare costs, difficult access, and poor quality of life (Shueb et al., 2015). Even though oral diseases display different symptoms, many patients seek dental advice due to mouth and facial pain (Badel et al., 2019) (Rotpenpian and Yakkaphan, 2021a).

The thermal and mechanical sensitivity of the oral mucosa differs from elsewhere in the body. The oral mucous membrane has Aδ-fibers and unmyelinated C-fibers innervations that are polymodal sensory afferents encoding nociception (Setty and David, 2014; Hossain et al., 2019). However, the trigeminal pain system has singularities (Setty and David, 2014). After a tissue injury, inflammatory mediators’ expression and proinflammatory cytokines are increased, activating the transient receptor potential (TRP) ion channels to provide the excitatory trigger in neurons (Bíró and Szallasi, 2012; Chung et al., 2011). Thermo-TRPs are temperature-dependent ion channels first described in the late ‘90s (Cesare and McNaughton, 1996; Caterina et al., 1997).

Several TRP channels are thermal sensors corresponding to a subgroup of ten channels that are expressed in sensory nerve endings and skin cells (Caterina, 2007). Recently, it was discovered that TRPC5 is essential for inflammatory tooth pain and, together with TRPA1, can explain cold tooth pain (Bernal et al., 2021). This emphasizes the crucial role of the TRP family in sensing orofacial pain (Luo et al., 2021). TRPV1 is the most popular member of the TRP family, and its ability to respond to capsaicin underscores the receptor as a pharmacological target (Rosenbaum and Simon, 2007). Understanding the capsaicin binding site and mechanism of action contributes to both the pharmacological and genetic basis of pain sensation (Julius, 2013). The bindings of agonist compounds are sufficient to produce conformational modifications in the channel, transforming the non-conducting “close state” to a conducting “open state” (Henderson, 2013). Experimental data suggest that most drugs, both agonists and antagonists, act on TRPV1 near the binding site of capsaicin (Yang and Zheng, 2017). TRPV1’s sensory activation and modulation by agonists and antagonists will improve our comprehension of how different molecular detectors -within a single ion channel protein-interact with the channel’s gate and propose new drug formulations (Carnevale and Rohacs, 2016). This review summarizes the principal advances in discovering new molecules targeting TRPV1 and their complex roles in signaling orofacial pain diseases.

## 2 TRPV1 involvement in inflammatory dental pain

**FIGURE 1:**
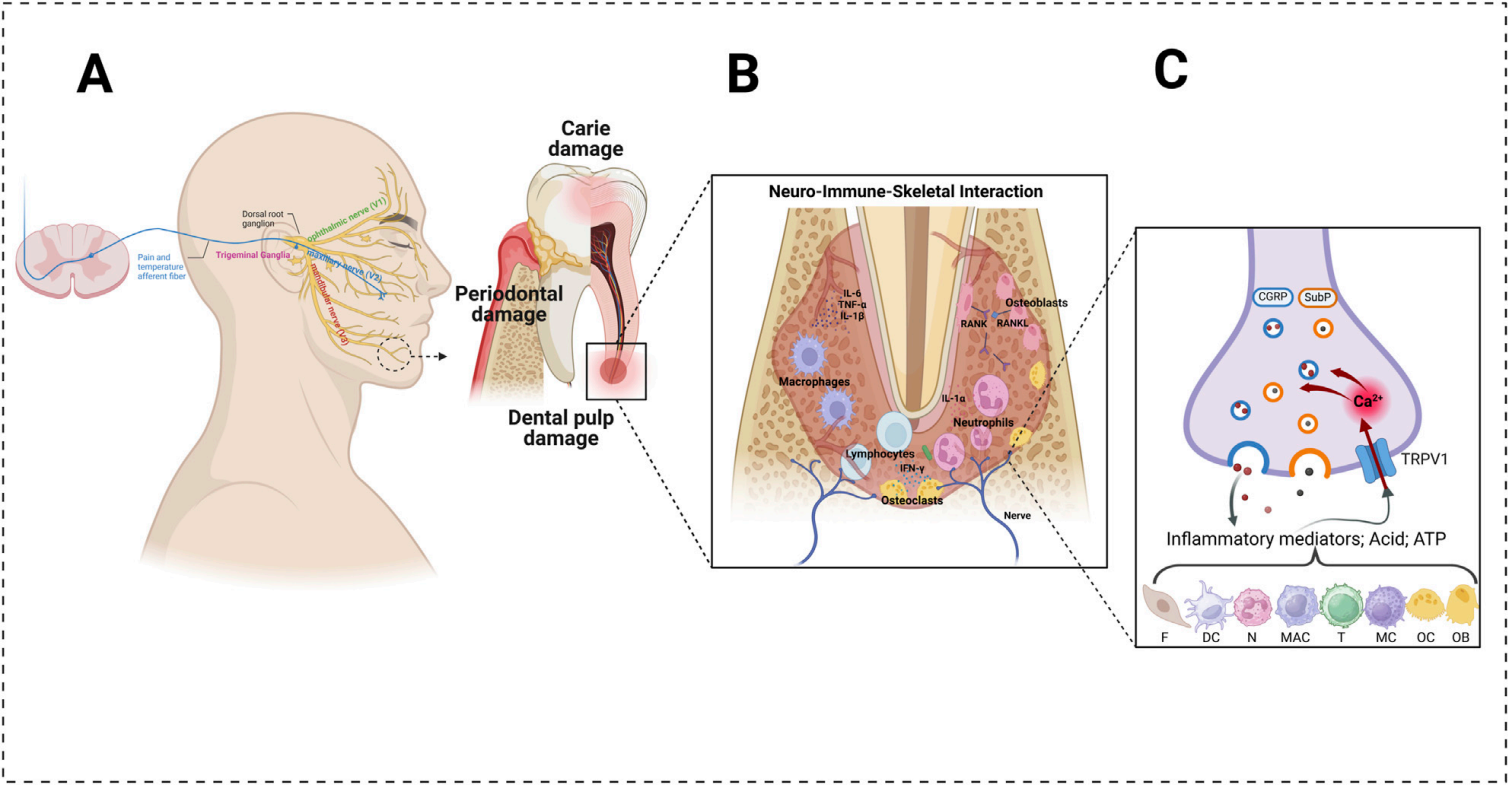
Involvement of the TRPV1 channel in the transduction of dental pain under inflammatory conditions. **(A)** Anatomy of the trigeminal afferents (left) and mandibular molar under the action of odontopathogenic factors (right). The alveolar bone undergoes resorption under infection. **(B)** In dental infection-inflammatory conditions, the various structures of the dentine–pulp–skeletal complex (e.g., odontoblasts, fibroblasts, dendritic cells, resident mast cells, neutrophils, monocytes, and T-lymphocytes) detect invading pathogens, leading to the initiation of an immune response. **(C)** Pro-inflammatory cytokines (e.g., interleukin-1β, tumor necrosis factor), inflammatory mediators (e.g., prostaglandin E2, bradykinin), and reactive oxygen metabolites (e.g., hydrogen peroxide, 8-hydroxy-2′-deoxyguanosine) can activate and sensitize the transient receptor potential vanilloid subtype 1 (TRPV1). Its activation induces calcium influx into nerve terminals and triggers the exocytosis of vesicles containing calcitonin gene-related peptide (CGRP) and substance P (SubP). The concerted activation of TRPV1 can generate an action potential, which is transmitted to the brain and leads to nociception. **(F)** fibroblast; DC: dendritic cell; MC: mast cell; MAC: macrophage; T: T-lymphocyte; N: neutrophil; OC: osteoclastic cell; OB: osteoblastic cell. Created with BioRender.com.

Dental caries is an ecological and non-communicable disease caused by acidic by-products from the bacterial fermentation of free sugars (Giacaman et al., 2022). It is the most common cause of orofacial pain caused by inflammatory reactions (Peres et al., 2019). If caries progresses and destroys hard tissue, it spreads to the pulp, causing inflammation, infection, and posterior tooth loss (Labanca et al., 2023b). Inflammation of the dental pulp or pulpitis is associated with thermal and mechanical hypersensitivity, for example, with the application of heat and cold stimuli (Gibbs et al., 2011a). Applying heat and cold stimuli evokes dental pain by activating the dental nociceptors in the dental pulp, which oversees the family of TRP channels (Gibbs et al., 2011b). The dental primary afferent neurons transmit the stimuli from the inferior alveolar nerve, a branch of the trigeminal nerve, specifically the mandibular branch (Rotpenpian and Yakkaphan, 2021a; Huff et al., 2023). After receiving excessive, uncontrollable inflammation due to pulpitis or a thermal stimulus, first-order neurons in the nerve will receive increased pain signals that arrive at the trigeminal ganglia and then are sent to the second-order neurons in the trigeminal nucleus caudalis. After that, the third-order neurons in the thalamus will receive the signals, and pain modulation occurs (Figure 1) (Rotpenpian and Yakkaphan, 2021a).

Recently, some investigators have discovered that TRP channels have been implicated in the transduction of external stimuli into pain signals, and these receptors have been detected in odontoblast and dental primary afferent neurons (Chung et al., 2011; Nilius and Szallasi, 2014). Gibbs et al. evaluated the percentage of rat trigeminal neurons that expressed TRPV1 and TRPV2 receptors; they observed that TRPV1 is expressed in 25% of the neuronal cell bodies in the trigeminal ganglia (TG) and TRPV2 in 15%. In the dental pulp, it is different; TRPV1 is expressed in 17% of the neurons and TRPV2 in about 50%, indicating that TRPV2 dominates the innervation compared with TRPV1 (Gibbs et al., 2011a). Another study suggests that TRPV1 is expressed in pulpal afferents in rat models (Chung et al., 2011). Furthermore, the expression of TRPV1 was observed in pulp inflammation evocated by pulp exposure; in the investigation of Cha et al., they observed that TRPV1 expression was significantly higher than the controls, which indicates that increased nociception signals in TG (Cha et al., 2020). Similar results have been observed by Chung et al., which established that applying lipopolysaccharide, a product of gram-negative bacteria, in the dentinal surface upregulates the expression of TRPV1 in mouse TG (Chung et al., 2011). Hossain et al. investigated the mechanism of the pain’s transduction. They observed that TRP channels act as polymodal receptors involved in the transduction of various external stimuli, and its mechanism consists of an external stimulus that activates and opens the TRP channels expressed in odontoblasts which produce the increase of intracellular Ca^2+^ concentration, so it transmits the nociceptor stimuli. Moreover, these odontoblasts can release ATP and glutamate, which act as agonists on the TRPV1 receptors of the adjacent nerve fibers of dental pulp afferents neurons and activate the sensory nerves of the dental pulp (Hossain et al., 2019). Purinergic receptors, primarily activated by ATP, and glutamatergic receptors, stimulated by glutamate, play a pivotal role in modulating TRPV1 activity. This influence can occur both directly, through dynamic interactions between receptors, and indirectly, by engaging complex intracellular signaling pathways (Jin et al., 2004).

Additionally, spontaneous pain in symptomatic pulpitis is due to the sensitization of dental pulp afferent neurons that produce hyperexcitability of the nerves (Hossain et al., 2019). This hyperexcitability is caused by the upregulation of the TRPs in these zones, which is seen in carious human teeth (Chung et al., 2011). This can also be explained because, under this inflammatory state, the threshold for the activation of TRP channels may decrease and cause hypersensitivity of the tooth to external stimuli (Hossain et al., 2019).

Some clinical studies have observed that TRPV1 acts as a mechanoreceptor in tooth pain, but it is controversial (De Petrocellis and Moriello, 2013). The influx of calcium through TRPV1 can significantly influence its activity, potentially modulating its sensitivity and interactions with other ion channels (Darré and Domene, 2015). Additionally, TRPV1 activation by low pH or TRPA1 activation can inhibit Piezo channels (Borbiro et al., 2015). This suggests that calcium influx through various pathways may modulate Piezo channel activity, highlighting the intricate crosstalk between these ion channels in sensory signaling. This mechanism explains that during inflammation, the extravasation of fluid from blood vessels can elevate the pulp pressure, which can excite the mechanoreceptors on the nerve fibers of the pulp and lead to spontaneous pain (Hossain et al., 2019).

## 3 Non-odontogenic orofacial pain

Orofacial pain (OP) is often associated with dental pathologies; nonetheless, the origin can be related to non-odontogenic disorders such as migraine, cancer, trigeminal neuralgias, and temporomandibular joint (TMJ) (Rotpenpian and Yakkaphan, 2021b). An estimated 25% of the population has experienced some form of OP, with the age range of 18–25 being the most common for this type of pain (Bender, 2018). The temporomandibular disorder includes the TMJ, masticatory muscles, and associated structures (Leite-Panissi et al., 2023a). Pain in the joint can be produced by disc disorders, diseases such as degenerative joint disease, systemic arthritis, osteonecrosis, neoplasm, and osteochondritis dissecans; fractures and congenital disorders can also produce it (Ahmad and Schiffman, 2016). Inflammation in the joint can lead to increased TRPV1, activated by heat, low pH, and capsaicin (Leite-Panissi et al., 2023a). It has been demonstrated that many TMJ TRPV-1 immunoreactive nerves are marked with a neuropeptide related to the calcitonin gene, which participates in nociception mechanisms and inflammation of this region (Leite-Panissi et al., 2023b) The activation of the TRPV1 channel can cause somatic inflammation both via peripheral sensitization and central sensitization (Park, 2015). There has been a great pharmaceutical effort to develop small-molecule inhibitors of TRP channels; however, there has been limited success (Park, 2015).

Another disorder that causes orofacial pain is trigeminal neuralgia (TN), which is described as a chronic neuropathic pain disorder characterized by recurrent, paroxysmal episodes of short-lasting severe intensity electric shock-like pain along the sensory distribution of the trigeminal nerve (Khawaja and Scrivani, 2023a). This type of pain causes a poor quality of life, and in some extreme cases, suicide has been attributed to the disorder (Cruccu et al., 2020). TN is a rare condition, with a prevalence of 0.03%–0.3%, and has an annual incidence of 4.3–8 per 100,000 with an average age of 53–57 years, with women the most affected by the disorder (Khawaja and Scrivani, 2023a). On the other hand, trigeminal neuropathic pain is associated with trigeminal nerve injury, which leads to chronic pain such as aching and burning (Urano et al., 2012a). It has been shown that TRPV1 afferents are involved in mechanical hyperalgesia in mice following a chronic constriction injury of the infraorbital nerve (Wang et al., 2020a). Inhibition of TRPV1 by injecting an antagonist of the channel attenuated mechanical hyperalgesia and ongoing pain (Wang et al., 2020a). Trigeminal neuropathic pain has also been associated with heat hyperalgesia, and as a form of treatment, capsazepine has been investigated, showing partial inhibition of heat hyperalgesia in a dose-dependent manner (Urano et al., 2012b).

As we mentioned before, OP can be caused by various non-odontogenic pathologies. One of them is cancer. Cancer is a disease that arises not from foreign substances invading our bodies but from the human cells, which transform into pathological organisms (Hausman, 2019). OP can be caused by regional or distant cancer (Romero-Reyes and Salvemini, 2016). Cancer in the orofacial region produces not only spontaneous pain but also affects the function of the region, having a negative impact on the quality of life of the patient (Romero-Reyes and Salvemini, 2016). The prevalence of pain in all cancer types is around 40%; nonetheless, pain in head and neck cancer has a prevalence of 70%, which increases to 90% in advanced cases (Khawaja and Scrivani, 2023b). TRPV1 channel has been linked to inflammation and calcium signaling (Bujak et al., 2019). It has been studied that chronic inflammation is associated with tumorigenesis, and aberrant calcium signaling promotes metastasis, proliferation, and cancer cell survival (Bujak et al., 2019). TRPV1 either promotes or inhibits cell death, depending on the type of cancer cell (Li et al., 2021). TRPV1 agonist/antagonist has been demonstrated to affect cell proliferation, cell death, and metastasis by activating TRPV1 channels and increasing intracellular calcium levels (Li et al., 2021). Migraine is another disease that causes OP, affecting more than 10% of the general population (Iannone et al., 2022a). TRPV1 contributes to this pain because it is expressed on dural nerve fibers of trigeminal origin in humans, which releases calcitonin gene-related peptide (CGRP) (Iannone et al., 2022b). CGRP is a member of the calcitonin family, in which levels in the cranial circulation are elevated during migraine attacks, and an intravenous infusion of CGRP causes migraine-like headaches. It has been demonstrated that capsaicin, a selective agonist of TRPV1, can be applied topically in the areas innervated by the trigeminal nerve, helping to improve the effects of cluster headaches and migraine (Dussor et al., 2014). This will occur due to capsaicin desensitization and, therefore, the reduced ability to release CGRP (De Logu et al., 2019a). TRPA1 is one of the primary transducers linked to the CGRP release, causing local inflammation, neuronal sensitization, and, therefore, pain (De Logu et al., 2019b).

Many patients who come in for a consult to treat pain in the orofacial region are wrongly diagnosed with toothache when they present a chronic OP condition (Renton, 2020). This can cause unnecessary surgery or giving wrong advice to the patient (Renton, 2020). To avoid inconsistent diagnosis, the clinician should first rule out odontogenic causes that could be causing the pain (Ando et al., 2022). Once this has been ruled out, the clinician should focus on non-odontogenic causes of OP, such as musculoskeletal, neurovascular, and neuropathic types of pain (Ando et al., 2022). It is essential that dentists maintain the possibilities open, expanding into examination techniques and consulting with another specialist if needed (De Laat, 2020). OP is an issue that is gaining more and more importance because it represents both a medical and social problem (Dubner et al., 2016). Inflammation causes the activation of many receptors on the terminals of afferent TG neurons, one of the main ones being TRPV1, which is expressed in orofacial tissues (Dubner, 2016). As mentioned before, TRPV1 is linked to many non-odontogenic disorders; it's because of this that it is important to keep all the possibilities open, knowing about all the possible causes of OP (De Laat, 2020).

## 4 Peripheral pathology

The international classification of orofacial pain mentions lesions or disorders affecting teeth and supporting areas as the main determinants of pain (Labanca et al., 2023c). Some viral infections of the gingival tissues can be ulcerated and painful to touch; this is increased by eating or drinking acidic, hot, or cold food or drinks, which also causes pain (Dubner, 2016). The oral mucosa also involves orofacial pain and implicated lesions such as ulcers, erosions, and vesicles (Rotpenpian and Yakkaphan, 2021b). One infection agent is herpes simplex virus (HSV). HSV is a neurotropic virus that becomes latent after infection, and it's located in the sensory ganglia (Kitagawa et al., 2013; Takasaki et al., 2000).

Kitagawa et al. (2013) demonstrated that herpes simplex virus (HSV) infection induces allodynia and hyperalgesia in a mouse model. To better understand the role of TRPV1 in arthritic and postherpetic pain, they examined the effects of JST-653, a selective TRPV1 antagonist. Their findings suggest that TRPV1 plays a significant role in the development of pain associated with these conditions, highlighting its potential as a therapeutic target for managing virus-induced and chronic inflammatory pain. Notably, the administration of JST-653 effectively attenuated mechanical hyperalgesia in the HSV-inoculated mice, providing strong evidence for TRPV1’s involvement in mediating pain hypersensitivity.

Orofacial pain can also be attributed to a lesion or disease of the cranial nerves (Rotpenpian and Yakkaphan, 2021b). The classification includes trigeminal neuropathic pain attributed to the varicella-zoster virus*;* it consists of a unilateral facial pain of less than 3 months duration that is distributed in one or more branches of the trigeminal nerve and is associated with clinical signs as acute herpes zoster (Classification of Chronic Pain, 2011). The reactivation of the varicella-zoster virus causes herpes zoster, and it causes acute herpetic pain (Takasaki et al., 2000). Sang et al. observed the expression of TRPV1 in skin herpes zoster lesions with immunofluorescent staining, which shows that it was increased and mainly present on epidermal keratinocytes compared with control skin, so they conclude that this receptor contributes to acute zoster pain and that there is a combination between nociceptive and neuropathic pain due to peripheral nerve sensitization (Han et al., 2016). Guedon & cols. inoculated rats with varicella-zoster virus, and through qPCR analysis of host gene expression, they observed a greater expression of TRPV1 in rats infected with the virus (Guedon et al., 2015). This is an important public health problem because only 30%-50% of patients can relieve their symptoms (Guedon et al., 2015).

Some traumas can cause chronic pain, and when they occur in the trigeminal nerve, they generate unilateral or bilateral facial persisting for more than 3 months (International Classification of Orofacial Pain, 2020). The origin of the trauma can be accidents, orthognathic surgery, or dental procedures (Kämmerer et al., 2024). It has been observed that TRPV1 plays an essential role in the development and maintaining of hyperalgesia induced by trigeminal neuropathic pain (J, 2005).

## 5 New molecules and possibilities

In clinical practice, various medications are used to treat pain, including opioids, nonsteroidal anti-inflammatory drugs (NSAIDs), antidepressants, antiepileptics, local anesthetics, muscle relaxants, and calcium channel blockers (Leeuw and Klasser, 2018; Khan et al., 2019a). However, these medications often provide unsatisfactory pain relief, may cause severe side effects, and can even lead to addiction (Furlan et al., 2006; Cavalli et al., 2019; Payne, 2000; Labianca et al., 2012). A logical approach to minimizing these side effects is to focus on peripheral pain receptors, where the pain originates. Among these receptors, TRPV1 stands out, as its activation can transmit and amplify pain through mechanisms that include increased neuronal excitability, the release of inflammatory mediators, and changes in neural plasticity (Gunthorpe and Szallasi, 2008). Therefore, TRPV1 has become an attractive target for developing new molecules to modulate this receptor (Jara-Oseguera et al., 2008).

Current studies are focused on molecules that act as agonists or antagonists of TRPV1 (LS and P, 2008; Premkumar and Sikand, 2008; Wong and Gavva, 2009). An agonist molecule initially binds to the receptor, activating it. This activation generates action potential, often perceived as itching, stinging, or burning sensations. However, when applied repeatedly or at high concentrations, it induces a long-lasting effect known as ‘desensitization’ of the TRPV1^+^ fibers. One example of a molecule used in this context is capsaicin (Arora et al., 2021). This compound has been utilized in clinical pain management, such as in cases of postherpetic neuralgia, HIV-related neuropathy, painful diabetic neuropathy, and chemotherapy-induced peripheral neuropathy (Derry et al., 2017).

A systematic review by Darry et al. (2017) evaluated the use of topical capsaicin for chronic neuropathic pain in clinical trials on postherpetic neuralgia, painful HIV-related neuropathy, and painful diabetic neuropathy. In cases of postherpetic neuralgia, patients reported feeling ‘much’ or ‘very much’ improved with high-concentration capsaicin (8%) compared to an ‘active’ placebo (0.04% topical capsaicin) after use for 8–12 weeks. In the case of painful HIV-related neuropathy, the studies included in the systematic review showed average reductions in pain intensity with the use of an 8% topical capsaicin patch for 2–12 weeks compared to the control. For painful diabetic peripheral neuropathy, the sole study reported 30% and 50% reductions in pain intensity over 2 to 8 and 2–12 weeks, respectively, when using 8% topical capsaicin compared to baseline (Derry et al., 2017).

Several studies have explored the use of capsaicin in orofacial pain management, mainly targeting conditions such as burning mouth syndrome (BMS)​ (Militão et al., 2021), oral neuropathic pain and trigeminal neuralgia (Epstein et al., 1994), oral mucositis (Berger et al., 1995a), and temporomandibular joint disorder (TMD) (Campbell et al., 2017)​. Ricken et al. (2021) demonstrated that a 180-day treatment with 0.025% capsaicin gel in BMS patients led to symptom reduction or complete remission and improved quality of life. In the study by Epstein et al. (1994), 31.6% of patients achieved complete remission of neuropathic pain, and an additional 31.6% reported partial remission. In contrast, topical treatments for trigeminal neuralgia showed no significant improvement. For oral mucositis, Berger et al. (1995b) (administered capsaicin in a candy (taffy) form, resulting in substantial pain relief for 11 patients undergoing cancer therapy. Regarding TMD, treatment with a high-dose (8%) capsaicin patch yielded significantly lower pain levels in TMD patients during the week following application compared to controls. Additionally, patients experienced a decreased thermal pain threshold 2 hours post-application, though it returned to baseline after 1 week.

On the other hand, TRPV1 antagonists block the receptor to relieve pain and itch sensations by inhibiting receptor activation and preventing nociceptive signals from traveling from peripheral sites to the central nervous system. Several novel small-molecule antagonists have been developed to prevent the activation of both native and heterologous TRPV1 channels (Table 1). These antagonists include BCTC, AMG9810, and SB-705498 (Gavva et al., 2005; Pomonis et al., 2003; Gibson et al., 2014). In orofacial pain studies, we identified two clinical trials that evaluated the antinociceptive activity of the molecules ABT-102 (Rowbotham et al., 2011a) and AZD1386 (Quiding et al., 2013a).

**TABLE 1:** Clinical trial orofacial pain therapeutic strategies targeting TRPV1.

TRPV1 targeted therapy	Author (Year)	Country	Compound	Administration route	Participant’s sex	Clinical data	Side effects
*TRPV1 chemical agonist*	Epstein et al. (1994)	Canada	Capsaicin	Topical: cream (0.025%)	19 female and 5 male	24 patients with oral neuropathic pain and trigeminal neuralgia. Complete pain remission in 31.6% of patients and partial remission in another 31.6%	Burning sensation at the site of application; some irritation and a disagreeable taste were reported​
	Berger et al. (1995)	USA	Capsaicin	Topical: candy (taffy vehicle) at a concentration of 0.025%	6 female and 5 male	11 patients with oral mucositis pain from cancer therapy. Pain reduction was substantial for most patients, with 9 out of 11 experiencing relief	Burning sensation upon initial application, which decreased with repeated use. No systemic side effects were reported
	Derry et al. (2017)	UK	Capsaicin	Topical: High-concentration (8%) topical capsaicin patch	911 female and 1,577 male	Involving 2488 participants across eight studies, the 8% capsaicin patch demonstrated moderate pain relief for postherpetic neuralgia, HIV-associated neuropathy, and diabetic neuropathy	Common localized reactions included redness, burning, and pain at the application site. Serious adverse events were rare and no more frequent than with control patches​
	Ricken et al. (2021)	Brazil	Capsaicin	Topical: capsaicin gel (0.025%)	18 female	The topical use of capsaicin gel was effective in reducing or remitting symptoms of BMS. Additionally, the treatment improved patients’ quality of life, as assessed by the OHIP-14 questionnaire, showing reduced discomfort and disability caused by BMS.	An initial increase in burning sensation was common but resolved with repeated applications. No systemic side effects were reported
	Campbell et al. (2017)	USA	Capsaicin	Topical: high-concentration (8%) capsaicin cream on TMD.	68 female	The study assessed topical capsaicin’s effects on 15 TMD patients and 53 healthy controls. TMD subjects reported significantly reduced pain a week after treatment. Two hours post-application, both groups showed decreased thermal pain thresholds. However, capsaicin did not affect pressure pain threshold or mechanical sensitivity in either group	Two participants (1 from each of the TMD and non-TMD capsaicin groups) requested the cream to be removed before the 2-h time limit after drug application due to excess pain
	ClinicalTrials.gov ID: NCT01886313 (2014)	United States	Civamide	Intranasal: Civamide Nasal Spray 0.01% 20ug/dose (20ul), 10ul in each nostril, twice daily, for 6 weeks	7 female and 4 male	Pain scores were measured from baseline to week 6, comparing Civamide (6 participants) to a placebo (5 participants). By week 6, pain scores decreased more in the placebo group (2.1-point reduction) than in the Civamide group (1.1-point reduction). However, the small sample size limits conclusions about treatment effectiveness	No serious adverse effects were observed. The most reported adverse effects associated with Civamide use were application site burning, application site irritation, sneezing, and pruritus
*TRPV1 chemical antagonist*	Rowbotham et al. (2011a)	USA, Denmark	ABT-102	Oral: at doses of 1 mg, 2 mg, and 4 mg twice daily	18 female and 18 male	The study was conducted with 36 healthy participants (18 men and 18 women) in a Phase 1 clinical trial, to evaluate the effects of ABT-102 on thermosensation and safety. Results showed that ABT-102 increased heat pain thresholds and reduced the perception of painful heat in a dose-dependent manner	ABT-102 had no effect on cutaneous cold detection and showed no significant safety concerns. During the first 24 h, mean temperature increases over placebo were 0.2°C (1 mg), 0.3°C (2 mg), and 0.6°C (4 mg). The highest recorded core body temperature was 38.7°C (day 1, 4 mg bid group)
	Quiding et al. (2013a)	Sweden, USA.	AZD1386	Oral: 95 mg solution	103 male	AZD1386 was tested after third molar extraction, compared to naproxen (500 mg) and placebo. It provided rapid pain relief (median onset: 0.3 h), faster than placebo (1.3 h) and similar to naproxen (0.8 h). However, it showed no significant difference in pain reduction over 8 h compared to placebo	Adverse events occurring more than once were dizziness, headaches, nausea and chills. The maximum increase in body temperature reported for patients treated with AZD1386 was 37.2°C (mean), recorded approximately 3 h after administration
	ClinicalTrials.gov ID: NCT00281684 (2005)	United Kingdom, Republic of Korea, and Italy	SB705498	Oral: SB705498 400 mg capsules, and SB705498 1,000 mg capsules	76 female and 69 male	Pain was assessed using VAS and VRS in four treatment groups: placebo (37 participants), SB705498 at 400 mg (36) and 1,000 mg (34), and Co-Codamol (38). Co-Codamol and SB705498 400 mg performed better compared to placebo and SB705498 1,000 mg	SB705498 was well-tolerated at the tested doses, with the highest frequency of side effects observed in the 1,000 mg group. The most common adverse effects included headache, fever, a sensation of warmth, and mild gastrointestinal symptoms. No treatment-related serious adverse events were reported

ABT-102 (Rowbotham et al., 2011a) was administered in doses of 4 mg, 2 mg, or 1 mg, given orally twice daily to male and female participants with no history of orofacial pain, oral trauma, or recent medication use that could interfere with the outcomes. Evaluations focused on the effects of ABT-102 on thermal sensation and induced pain. Assessments included quantitative thermosensory testing (QTT) performed on the abdominal skin and tongue, an oral liquid test (OLT) using heated water, prolonged thermal stimulation (LTS), and a hot water bath test (WBT). Additionally, adverse events such as core body temperature, vital signs, and laboratory tests were monitored. The main results from the Phase 1 clinical study indicated dose-dependent modulation of thermosensation, particularly in parameters such as the heat pain threshold (HPT) and OLT. ABT-102 significantly increased heat pain thresholds in both cutaneous and oral regions at all tested doses (1 mg, 2 mg, and 4 mg, twice daily), with the most pronounced effects observed 5 h after administration.

Regarding safety, adverse events were reported by 70.8% of participants receiving ABT-102, compared to 58.3% in the placebo group. The most common adverse events included increased body temperature, headache, nausea, feeling hot, and abdominal discomfort, all classified as mild or moderate. No serious adverse events, deaths, or clinically significant laboratory, ECG, or vital sign parameter changes were observed (Table 1) (Rowbotham et al., 2011b).

AZD1386 (Quiding et al., 2013b) was evaluated for its antinociceptive activity in patients undergoing surgical removal of at least one impacted mandibular third molar and, when indicated, the ipsilateral upper third molar. After surgery, patients who requested pain relief within 6 h were randomized to receive one of three treatments: 95 mg AZD1386 (solution), placebo (solution/capsule), or 500 mg naproxen (capsule). AZD1386 demonstrated rapid and significant pain relief compared to placebo, particularly in the first few hours after administration. Pain relief onset was faster with AZD1386 (median of 0.3 h for first perceptible relief) compared to placebo (1.3 h) and naproxen (0.8 h). Although AZD1386 showed efficacy in early pain relief measures (pain intensity difference PID% within the first hour), the primary outcome (weighted pain intensity difference over 8 h SPID%) was not significantly different from placebo. Approximately 85% of patients treated with AZD1386 reported perceptible pain relief, but about 80% still required rescue analgesics, comparable to the placebo group. Naproxen demonstrated superior efficacy to AZD1386, with fewer patients needing rescue analgesics (48%).

Regarding safety, there were no early discontinuations or serious adverse events. A slight increase in body temperature was observed with AZD1386 use (maximum of 38.1°C in two patients), but it had no significant clinical impact. Additionally, laboratory and ECG parameters showed no clinically relevant changes (Table 1) (Quiding et al., 2013b).

Civamide (cis-8-methyl-N-vanillyl-6-nonenamide), a TRPV1 agonist and neuronal calcium channel blocker, has been studied for orofacial pain management due to its ability to inhibit the neuronal release of excitatory neurotransmitters acting as a calcium channel blocker. A clinical trial (ClinicalTrials.gov ID: NCT01886313) (Study Details | Civamide Nasal) registered in March 2014 evaluated Civamide Nasal Spray 0.01% [20 µg/dose (10 µL per nostril, twice daily)] for 6 weeks in patients diagnosed with postherpetic neuralgia of the trigeminal nerve. The trial included 11 participants: 6 in the Civamide group and 5 in the placebo group. At baseline, mean pain scores were 6.4 (±0.84) for the Civamide group and 5.2 (±1.02) for the placebo group; by week 6, scores instantly decreased to 5.3 (±1.24) and 3.1 (±2.13), respectively. Thus, the small sample size limited the study’s statistical power to detect significant differences between groups. The more frequently reported adverse effects were site burning, irritation, sneezing, and pruritus after Civamide use.

The study (ClinicalTrials.gov ID: NCT00281684) used a third molar extraction model to test the efficacy of the TRPV1 antagonist SB705498 in controlling pain after surgery. A total of 145 participants were divided into four groups: placebo (n = 37), SB705498 at 400 mg (n = 36) and 1,000 mg (n = 34), and co-codamol (n = 38). Pain intensity was assessed using the visual analog scale (VAS) and verbal rating Scale (VRS) over 10 h post-treatment. The data from VAS demonstrated that co-codamol and SB705498 400 mg outperformed better than placebo and SB705498 1,000 mg. VRS scores supported these findings, with Co-Codamol showing the most remarkable efficacy (0.5 ± 1.18), followed by SB705498 1,000 mg (0.6 ± 0.53), SB705498 400 mg (0.8 ± 1.04), and placebo (1.1 ± 0.83). SB705498 was well-tolerated for both doses, with the highest frequency of side effects in the 1,000 mg group. Common adverse effects included headache, fever, warmth sensation, and mild gastrointestinal symptoms. The authors reported no serious treatment-related adverse effects.

Thus, preclinical studies investigating new molecules targeting TRPV1 to treat orofacial pain are presented here (Table 2) (Arora et al., 2021; Bai et al., 2018; Santos et al., 2022; Park et al., 2009; Melo et al., 2017; de Oliveira et al., 2022a; Guo et al., 2019; Gualdani et al., 2015; Hitomi et al., 2016; Kun et al., 2012; de la Rosa-Lugo et al., 2017; Damasceno et al., 2016; Wang et al., 2020b; Biggs et al., 2008a; Ando et al., 2020; Yeon et al., 2010; Wu et al., 2010; Anderson et al., 2014; Soares et al., 2019; Wanasuntronwong et al., 2022; Neubert et al., 2008; Lee et al., 2018; Bai et al., 2021; Rocha et al., 2022a). It identified 24 studies that used different animal models, with rats (n = 14) and mice (n = 9) being the most frequently employed species. The Zebrafish model for nociception evaluation was tested in a few (n = 3) studies, but it is considered a recent model but may answer a variety of tools to study pain and associated behavior (Leite-Panissi et al., 2023b; de Oliveira et al., 2022b; Rocha et al., 2022b). One (n = 1) study evaluated the efficacy of SB-750364 TRPV1 antagonist on injury-induced discharge in the lingual nerve in a ferret model. The data revealed a spontaneous activity reduction in fine filaments from terminal nerves in 61% (Biggs et al., 2008b).

**TABLE 2:** Preclinical studies on TRPV1-targeted treatments for orofacial pain.

Author	Country	Compound	Species	Treatment groups	Pain induction	Evaluation sites	Main results
Barreto et al. (2022)	Brazil, Canada	Botulinum toxin type A	Zebrafish	BoNT/A (0.05, 0.1, and 0.5 IU/masseter) vs vehicle	Cinnamaldehyde (0.66 μg/mL); capsaicin (40.93 μL/mL); menthol (1.2 μmol); glutamate (12.5 mM); or acid saline (0.1%)	Lip and masseter	Pretreatment with BoNT/A reduced nociceptive behavior induced by capsaicin and glutamate. Antinociception was effectively inhibited by capsazepine and ketamine, as well as by capsaicin-induced desensitization
Gualdani et al. (2015)	USA	ADM_12	Rats	ADM_12 (30 mg/kg, p.o.) vs vehicle	Complete Freund’s Adjuvant (CFA) injection for inflammatory sensitization of the trigeminal nerve	Orofacial region (mechanical facial allodynia)	ADM_12 demonstrated a significant reduction of mechanical facial allodynia induced by CFA injection, indicating potent antiallodynic effects. ADM_12 acted as a dual antagonist for TRPA1 and TRPV1, blocking currents induced by TRPA1 and TRPV1 agonists *in vitro*. ADM_12 also showed a favorable safety profile, with no cardiotoxicity observed at concentrations up to 1 mM
Damasceno et al. (2016)	Brazil	Frutalin (FTL)	Mice and rats	FTL (0.25, 0.5, and 1 mg/kg) vs vehicle	formalin (1%, 20 μL, perinasal); capsaicin (20 μL, 2.5 mg, s.c.); glutamate (20 μL, 25 mM); hypertonic saline (5 M NaCl, corneal nociception)	Orofacial region, temporomandibular joint (TMJ), cornea	Pretreatment with FTL significantly reduced nociceptive behaviors induced by formalin, capsaicin, and glutamate. L-NAME and D-galactose inhibited the antinociceptive effect but not by naloxone, suggesting the involvement of the nitrergic system rather than the opioid system. FTL was also effective in reducing cold-induced hyperalgesia after infraorbital nerve transection
Leite et al. (2022)	Brazil	Parkia platycephalic Lectin (PPL)	Zebrafish, mice, and rat	PPL (0.025, 0.05, 0.1 mg/mL) vs vehicle	Cinnamaldehyde (0.66 µg/mL), glutamate (12.5 µM), acid saline (0.1%), hypertonic saline (5 M NaCl), and infraorbital nerve transection	Orofacial region (zebrafish lips), temporomandibular joint (TMJ), and corneal surface	PPL significantly reduced nociceptive behaviors in models of acute and neuropathic orofacial pain. Effects were mediated by TRPV1 modulation, as shown by inhibition with capsazepine and capsaicin-induced desensitization
Soares et al. (2019)	Brazil	Oleanolic acid	Zebrafish	Oleanolic acid (0.1, 0.3, 1.0 mg/mL, oral) vs vehicle vs morphine (5 mg/mL)	Formalin (0.1%), cinnamaldehyde (0.33 μM), capsaicin (40.93 μM), menthol (1.2 mM), acidic saline (0.1%), glutamate (12.5 μM), and hypertonic saline (5 M NaCl)	Orofacial region (zebrafish lips)	Oleanolic acid reduced acute nociceptive behaviors in multiple pain models. Antinociception was mediated by TRPV1, as demonstrated by inhibition with ruthenium red and capsaicin-induced desensitization. Molecular docking studies suggested direct interaction with TRPV1
Ando et al. (2020)	Japan	Oxytocin (OXT)	Rat	Oxytocin (1.0 × 10^−6^ mol, 1.0 × 10^−8^ mol) vs vehicle; Oxytocin receptor antagonist (atosiban)	Infraorbital nerve injury (IONI) via ligation of one-third of the infraorbital nerve	Whisker pad skin	Oxytocin attenuated the orofacial mechanical allodynia after infraorbital nerve injury. It regulated TRPV1 and TRPV4 channel expression in trigeminal ganglion neurons. The antinociceptive effects of oxytocin were reversed by co-administration with atosiban​
Yeon et al. (2010)	Korea	Curcumin	Rats	Curcumin (5, 25, 50 mg/kg) vs. TRPV1 antagonist vs. vehicle	Subcutaneous capsaicin injections in the orofacial area	Trigeminal system, vibrissa pad	Curcumin dose-dependently blocked capsaicin-induced TRPV1 activation, reducing thermal hyperalgesia. The vanilloid moiety in curcumin is key for TRPV1 antagonism
Wu et al. (2010)	China	17-beta-estradiol	Rats	Control, sham ovariectomy, ovariectomized (OVX) with doses of 17-β-estradiol (0, 20, 80, 200 μg, s.c.).	Injection of 50 μL of CFA into the bilateral TMJ.	Hippocampus, TMJ.	17-β-estradiol replacement increased TRPV1 expression in the hippocampus and enhanced mechanical allodynia associated with TMJ inflammation, suggesting central modulation by TRPV1 in pain processing
Neubert et al. (2008)	USA	Resiniferatoxin (RTX)	Mouse	resiniferatoxin (RTX, 20 μL, intracisternal)	Thermal stimulus (37°C–55°C) and capsaicin (corneal application)	Orofacial region and cornea	The elimination of TRPV1-expressing neurons by RTX blocked nociceptive responses to heat and capsaicin, highlighting the role of TRPV1 in thermal and neurogenic pain
Anderson et al. (2014)	USA	Menthol and capsaicin	Rat	Menthol (1.25%), capsaicin (0.05%), and their combination	Thermal stimulation at 33°C, 21°C, and 45°C using the Orofacial Pain Assessment Device (OPAD)	Orofacial region (via OPAD)	Co-application of menthol and capsaicin reduced nociceptive responses to cold stimuli (21°C) compared to individual applications, highlighting selective modulation of TRP channels
Santos et al. (2022)	Brazil	Citral	Mice, Rats	pretreatment with citral (0.1, 0.3, or 1.0 mg/Kg, p.o.) vs. vehicle (control)	2% formalin, cinnamaldehyde (0.66 μM, 20 μL), menthol (20 μL, 1.2 mM), capsaicin (2.5 μg), mustard oil (20%; 20 μL)	Lip, temporomandibular joint (TMJ), masseter muscle, infraorbital nerve transection	Citral significantly reduced nociceptive behaviors in acute and chronic pain models in the orofacial region. Antinociception was mediated by TRPV1, TRPM3, and TRPM8, but not by TRPA1
Bai et al. (2021)	China	Stem cells	Rats	SHED (local: 1 × 10^5^ and 1 × 10^6^ cells; systemic: 1 × 10^5^ cells) vs. vehicle	Chronic constriction injury of the infraorbital nerve (ION-CCI)	Trigeminal ganglion, trigeminal nerve, injury site	SHED reduced mechanical sensitivity, inflammatory cell infiltration, and TRPV1 expressions, with effects lasting 8 weeks. Results support SHED as a potential therapy for trigeminal neuralgia
Guo et al. (2019)	China	Lentivirus containing a TRPV1 shRNA sequence	Rats	Orthodontic force alone, orthodontic force combined with TRPV1 antagonist SB366791 Orthodontic force combined with saline group Orthodontic force combined with TRPV1 shRNA lentivirus Orthodontic force combined with blank lentivirus group	Orthodontic force-induced tooth movement	Trigeminal ganglion, periodontal tissues	TRPV1 expression increased with orthodontic force TRPV1 antagonist and lentivirus-mediated gene therapy reduced TRPV1 levels and alleviated pain, demonstrating a therapy strategy for orthodontic pain management
Arora et al. (2021)	USA	Capsaicin	Mice	Capsaicin (single injection) vs. paclitaxel + capsaicin	Chronic constriction injury of the infraorbital nerve (ION-CCI)	Facial skin, infraorbital nerve	Capsaicin-induced microtubule depolymerization led to long-lasting analgesia, while paclitaxel inhibited axonal ablation and analgesic effects, showing the structural role of microtubules in TRPV1-mediated analgesia
de Melo Júnior et al. (2017)	Brazil	Eucalyptol	Mice	Control (vehicle) and eucalyptol (100, 200, 400 mg/kg)	Formalin (1%, 20 μL, perinasal), capsaicin (20 μL, 2.5 μg, perinasal), glutamate (20 μL, 25 mmol), hypertonic saline (5 M NaCl, corneal application). Neuropathic pain was induced by infraorbital nerve transection (IONX)	Orofacial region, temporomandibular joint (TMJ), cornea	Eucalyptol significantly reduced acute and neuropathic nociceptive behaviors, possibly acting as a TRPV1 channel antagonist
Bai et al. (2018)	China	Testosterone	Rat	Control males, orchiectomized (ODX) males, and ODX males treated with testosterone replacement (16 mg/kg, s.c.).	50 μL injection of 50% Complete Freund’s Adjuvant (CFA) into the left masseter muscle	Trigeminal ganglia (TG)	TRPV1 expression in the TG was significantly increased following inflammatory pain induction in ODX rats but not in testosterone-treated rats. This suggests that testosterone inhibits TRPV1 upregulation, modulating inflammatory hyperalgesia
de la Rosa-Lugo et al. (2017)	Mexico	Alkamides	Mice	Natural alkamides (1–30 µg) and synthetic alkamides (0.1–100 µg)	Orofacial formalin test in mice (2% formalin)	Orofacial region	Both natural and synthetic alkamides induced significant antinociception. The effects were mediated by TRPV1, as demonstrated by antagonism with capsazepine. Alkamides showed potential for the development of new analgesic drugs
Wang et al. (2020a)	USA	Capsaicin	Mice	Capsaicin (10 μg, s.c.), administered alone or with MDL28170 (a calpain inhibitor), compared to the vehicle	Chronic constriction injury of the infraorbital nerve (ION-CCI) in a mouse model	Facial skin in the infraorbital (V2) and mandibular (V3) regions	Capsaicin induced long-lasting analgesia, reducing mechanical hyperalgesia in trigeminal neuropathy models through selective ablation of TRPV1 afferent terminals. Co-administration of MDL28170 blocked these effects, implicating calpain activation in the analgesic mechanism
Kun et al. (2012)	Hungary	Resiniferatoxin (RXT)	Rat	RTX (30, 70, and 100 µg/kg, subcutaneous) vs saline (control)	Chemical and surgical denervation in rat hind limbs	Dorsal paw skin, plantar skin, and oral mucosa	RTX selectively desensitized neural TRPV1 receptors, reducing neurogenic inflammation. Non-neural TRPV1 expression remained unaffected, confirming the specificity of RTX for neural TRPV1 receptors​
Biggs et al. (2008a)	UK	SB-750364	Ferrets	SB-750364 (0.3, 1.0, and 3.0 µM) administered systemically via the cephalic vein	Lingual nerve injury (nerve sectioning) in ferrets, followed by spontaneous ectopic discharge recordings	Lingual nerve	SB-750364 significantly reduced spontaneous ectopic activity in 61% of units at 1.0 µM and 3.0 µM, indicating its effectiveness as a TRPV1 antagonist for relieving neuropathic pain​
Park et al. (2009)	Korea	Eugenol	Rats	Eugenol (50, 100, 200 µg) vs. control	Thermal stimulation (infrared in the vibrissal area) and capsaicin (5 μg, s.c.).	Orofacial region (vibrissal area), inferior alveolar nerve	Eugenol significantly reduced thermal nociception and capsaicin-induced thermal hyperalgesia in a dose-dependent manner, inhibiting action potential conduction in nociceptive nerves
Hitomi et al. (2016)	Japan	Hangeshasinto	Rats	Hangeshashinto (100 mg/mL, topical), lidocaine (1%), and indomethacin (5 mg/kg, intraperitoneal)	Oral ulcers induced by 50% acetic acid applied to the oral mucosa of rats	Labial fornix region of the oral mucosa	Hangeshashinto significantly reduced mechanical hypersensitivity and spontaneous pain in ulcerated oral mucosa without affecting healthy mucosa. Indomethacin reduced spontaneous pain but failed to block mechanical hypersensitivity​
Lee et al. (2018)	Taiwan	GTP, memantine, GTPm	Mice	Memantine (10 mg/kg), Green Tea Polyphenols (GTP, 60 mg/kg), GTPm (combination of GTP 30 mg/kg with memantine 3 mg/kg), and morphine (7.5–30 mg/kg) with/without memantine or GTPm, compared to the vehicle	Capsaicin (10 mg/5 μL, s.c.) in the vibrissa pad	Orofacial region and hind paw (hot plate test)	GTPm significantly reduced pain behaviors induced by capsaicin and enhanced the analgesic effects of morphine while reducing its depressive side effects and delaying morphine tolerance
Wanasuntronwong et al. (2022)	Thailand	Cannabidiol	Mice	Cannabidiol (0.5, 1, 3, 5, and 10 mg/kg) with/without CGRP or vanilloid receptor 1 (VR1) antagonists, compared to control (saline)	CFA (10 µL) was injected into the right masseter muscle	Masseter muscle, trigeminal nucleus caudalis, periaqueductal gray, and raphe magnus nucleus	Cannabidiol showed dose-dependent antinociceptive effects mediated by the vanilloid receptor 1 (VR1) but not by CGRP. It reduced c-fos expression in nociception-related regions, suggesting its potential as an alternative or synergistic pain treatment

**FIGURE 2:**
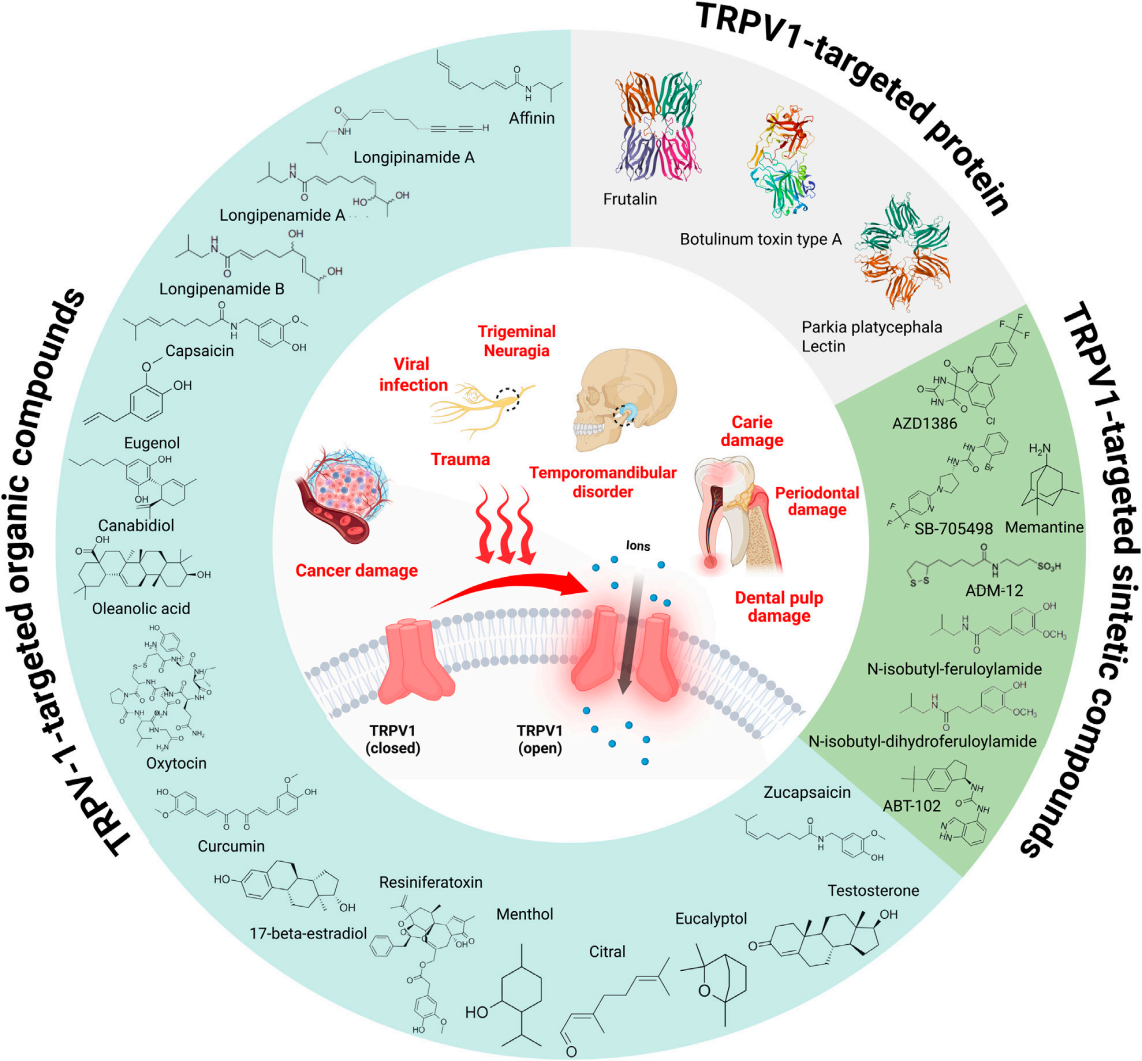
Schematic representation of orofacial factors contributing to TRPV1 activation (inner circle) alongside chemical and protein molecules studied in the articles included in this review. Chemical structures were drawn using Marvin - Chemical Drawing Software, while protein structures were retrieved from the RCSB Protein Data Bank (RCSB PDB). The figure was created with BioRender.com.

Among the molecules investigated, capsaicin and resiniferatoxin (RTX) were extensively evaluated, appearing in multiple studies (2 articles each). Most of the substances tested were of natural origin, such as eucalyptol, oleanolic acid, curcumin, menthol, capsaicin, eugenol, and cannabidiol, reflecting the interest in bioactive plant compounds and their antinociceptive properties. In addition, synthetic compounds such as ADM_12 and SB-750364 and emerging therapies, including stem cells derived from dental pulp (SHED) and Lentivirus containing a TRPV1 shRNA sequence, were also addressed, exploring new intervention possibilities for orofacial pain. Pain induction models varied from chemical stimuli, such as capsaicin, formalin, and glutamate, to neuropathic models involving nerve injuries or transections, such as chronic infraorbital nerve constriction. Most studies reported promising results, demonstrating that the evaluated molecules significantly reduced nociceptive or hyperalgesia behaviors, modulating the TRPV1 receptor. These data highlight the potential of new therapeutic approaches for orofacial pain conditions mediated by this receptor. Figure 2 shows all molecules and proteins studied in the articles included in this review.

## 6 Review highlights and discussion

Patients undergoing orofacial pain often face significant expenses, averaging £333 per 6-month period, including treatment fees, medication, and travel costs. Furthermore, the indirect costs to employers are even more pronounced, with absenteeism contributing an average of £1,242 per person over the same period (Breckons et al., 2018). Recent findings also emphasize the variability of financial impacts based on the severity of persistent orofacial pain. Patients classified with high-graded chronic pain scale (GCPS) status experience a £366 increase in healthcare costs within 6 months compared to those with lower GCPS levels (Durham et al., 2016). These findings underscore the multifaceted impact of orofacial pain, which extends beyond physical suffering to include significant social consequences.

Diagnosing and managing chronic orofacial pain remains a significant clinical challenge due to its multifaceted nature and the often-ambiguous overlap of symptoms with other conditions (Classification of Chronic Pain, 2011; Diatchenko et al., 2005). Chronic orofacial pain is notably complex because of its multifactorial etiology (Classification of Chronic Pain, 2011). As previously discussed, this type of pain can stem from various underlying issues, including inflammatory dental pain, non-odontogenic orofacial pain, and peripheral pathologies (Giacaman et al., 2022; Khan et al., 2019b).

Currently, one of the most pressing challenges in orofacial pain management lies in the use of medications (Abd-Elsayed et al., 2024). Despite advancements, the limitations in pain control are evident, with a notable incidence of adverse reactions that pose substantial risks to patients (Häggman-Henrikson et al., 2017). The use of analgesics, for example, is fraught with concerns regarding addiction, tolerance, and hypersensitivity reactions (Furlan et al., 2006; Cavalli et al., 2019; Payne, 2000; Labianca et al., 2012). Furthermore, these medications often result in additional side effects, such as gastrointestinal bleeding and an increased risk of cardiovascular events (Cavalli et al., 2019; Porreca and Ossipov, 2009). Anti-inflammatory therapies, while commonly employed, frequently offer only limited relief, necessitating supplementary treatments to achieve satisfactory pain management (Hersh et al., 2020). Given these challenges, there is a growing interest in targeted therapeutic approaches aimed at addressing the root cause of pain. Emerging research focuses on identifying molecules that can directly modulate nociceptive pathways, particularly those involving terminal regions where pain originates (Gunthorpe and Szallasi, 2008). A promising study area involves targeting receptors such as TRPV1, which are implicated in the transmission and modulation of pain signals (Wang et al., 2020b; Zhang et al., 2021; Luo et al., 2018). These novel strategies hold the potential to overcome the limitations of traditional therapies, offering a more precise and practical approach to managing chronic orofacial pain (Rotpenpian and Yakkaphan, 2021b; Dubner, 2016).

According to the articles in this review, the various molecules studied with TRPV1 activity demonstrated antinociceptive activity in preclinical and clinical trials (Jara-Oseguera et al., 2008). However, during these tests, adverse effects on body temperature (Tb), primarily hyperthermia, were repeatedly observed (Rowbotham et al., 2011b; Quiding et al., 2013c). Due to these side effects, clinical trials and the subsequent advancement toward the commercialization of these drugs have been delayed or halted (De Petrocellis and Moriello, 2013). One potential solution is to develop molecules that act on TRPV1 with sufficient analgesic efficacy but without altering Tb, such as compounds that do not block the activation of hTRPV1 by protons and temperature but remain potent blockers of capsaicin (CAP) or those with low blocking potency for protons and heat while maintaining high CAP-blocking potency (Garami et al., 2020). Another alternative is the combination of TRPV1 antagonists with antipyretic drugs, though this approach requires further research, as the temperature alteration linked to TRPV1 is not mediated by cyclooxygenase (Engström et al., 2013). A promising alternative might involve blocking sympathetic activation, as Garami et al. (2020) suggested.

Another point worth discussing is the predominant use of male animals in research studies (Santos et al., 2022; Park et al., 2009; Guo et al., 2019; Hitomi et al., 2016; Kun et al., 2012; Ando et al., 2020; Yeon et al., 2010; Anderson et al., 2014; Wanasuntronwong et al., 2022; Lee et al., 2018; Bai et al., 2021; Hwang et al., 2020). This approach raises controversy, given that women report orofacial pain more frequently than men (Häggman-Henrikson et al., 2020). Orofacial pain is approximately twice as prevalent in adult women (20–70 years) compared to men in the same age range, with an odds ratio (OR) of 2.58 (95% CI: 2.48–2.68). Furthermore, women exhibit a significantly higher risk of developing orofacial pain (OR 2.37) and persistent pain (OR 2.56) (Häggman-Henrikson et al., 2020). Temporomandibular disorders (TMD) are also more common in women, who are 2.2 times more likely to experience them (OR = 2.24).

Regarding specific diagnoses, women are 2.08 times more likely to develop muscular disorders, while the prevalence of disc displacement is 1.6 times higher in women. Additionally, conditions such as arthralgia, arthritis, or arthrosis are 2.09 times more likely to occur in women than in men (Bueno et al., 2018). These findings underscore the importance of considering gender differences in future studies, as data obtained exclusively from men cannot be validly generalized to women. Although several molecules with antinociceptive activity targeting TRPV1 have been proposed to manage orofacial pain, some limitations still prevent these molecules from being effectively made available to the population. Therefore, further studies are needed to understand their mechanisms of action better, minimize adverse effects such as alterations in body temperature, and ensure their long-term safety and efficacy.

## 7 Conclusion

In summary, we have reviewed a large body of evidence on TRPV1 in orofacial pain, mainly focused on the advances in discovering new molecules targeting TRPV1 and their contributions to pain behaviors. Orofacial pain is one of the most debilitating human diseases and represents a social and economic burden for our society. Managing orofacial pain is still a significant challenge for healthcare professionals, and the potential for work-related disability further contributes to a considerable economic strain on the healthcare system.
